# Mental health status of Italian elderly subjects during and after quarantine for the COVID‐19 pandemic: a cross‐sectional and longitudinal study

**DOI:** 10.1111/psyg.12703

**Published:** 2021-05-06

**Authors:** Gianpaolo Maggi, Ivana Baldassarre, Andrea Barbaro, Nicola Davide Cavallo, Maria Cropano, Raffaele Nappo, Gabriella Santangelo

**Affiliations:** ^1^ Department of Psychology University of Campania “Luigi Vanvitelli” Caserta Italy

**Keywords:** cognitive failures, COVID‐19, elderly, longitudinal changes, mental health, quarantine

## Abstract

**Background:**

The elderly are more vulnerable to COVID‐19 and therefore need to adopt long‐term social distancing measures. The duration of quarantine impacts the psychological status of the general population. However, until now no study has explored the psychological impact of the pandemic and quarantine together with longitudinal changes in the mental health status of Italian elderly.

**Methods:**

An online questionnaire including an assessment of depression, anxiety, anger, post‐traumatic stress, subjective cognitive failures, resilience, coping style, and other dimensions related to the pandemic was completed by participants during (T0) and two months after the end (T1) of the quarantine.

**Results:**

The sample recruited at T0 included 334 elderly participants. About 45% of the participants experienced depression, anxiety, or anger. Moreover, more fear of getting infected was related to more severe depression, anxiety, and anger, but resilience was found to mediate these relationships. More severe depressive and anger symptoms were related to more severe cognitive failures. No significant difference was observed in mental health scores between T0 and T1.

Finally, more severe depression at T0 was associated with the development of post‐traumatic stress symptoms at T1.

**Conclusions:**

The fear of getting infected, probably due to perceived vulnerability to disease, seems to play a crucial role in the development of psychological symptoms in the elderly, but resilience seems to mediate the impact of fear. The presence of long‐term psychological consequences and the possible risk of developing PTS symptoms in the elderly suggest the need for targeted interventions to reduce possible long‐term psychological and cognitive consequences.

## INTRODUCTION

The pandemic caused by the novel coronavirus disease (COVID‐19) caused an unprecedented social and health crisis all over the world. In addition to fear and suffering caused by the pandemic, the stay‐at‐home restrictions, quarantines, and lockdowns imposed by governments of different countries to control the spread of the virus could negatively impact the mental health status of individuals.^1^

A recent review^1^ exploring the psychological impact of quarantine during previous outbreaks showed more severe post‐traumatic stress (PTS) symptoms, depression, anger, and emotional exhaustion in individuals who were quarantined compared to those not quarantined.^2^, ^3^, ^4^, ^5^, ^6^

As for the psychological response to the COVID‐19 pandemic, in China where the outbreak started, 53.8% of respondents in an online survey reported moderate to severe psychological impact, including moderate to severe depressive symptoms (16.5%), anxiety (28.8%), and stress levels (8.1%).^7^ Moreover, the prevalence of anxiety and depressive symptoms in people affected by quarantine was double compared to unaffected individuals.^8^

Concomitantly with the spread of the COVID‐19, psychological symptoms were reported also in European populations. More specifically, more severe depressive and anxiety symptoms and higher stress levels were reported in Italian,^9^, ^10^ Spanish,^11^, ^12^ Turkish,^13^ and UK general populations.^14^

However, while the above‐mentioned studies explored the psychological consequences of the outbreak and quarantine/self‐isolation on the general population, the effects on the mental health status of more vulnerable groups such as the elderly population in particular deserves to be investigated. Indeed, elderly people are characterised by unique physical, psychosocial, and environmental vulnerabilities^15^ and they may be more at risk from COVID‐19 than people of other ages due to a fragile immune system and chronic comorbidities.^16^, ^17^ Therefore, the elderly population needs to practice social distancing limiting their interactions even with family members, and this could increase the loneliness and anxiety due to quarantine/self‐isolation and the uncertainty and fear due to the outbreak. Thus, restrictive measures could contribute to the rise of psychological symptoms such as depression, anxiety, anger, and subjective cognitive failures.

Previously, Meng *et al*.^16^ explored the psychological impact of COVID‐19 outbreak amongst the elderly population in China, revealing that 37.1% of participants experienced depression and anxiety. Moreover, the authors suggested focusing on female elderly, low educated elderly, those who are living alone, and those having mental health issues, recommending the implementation of psychological interventions to control the severity of their psychological symptoms.

Further, some studies investigating the psychological consequences of the COVID‐19 pandemic and self‐isolation measures through follow‐up methodologies have broadly explored individuals' mental health status during the early phase and the peak of the COVID‐19 outbreak or at the start and the end of the lockdown. Otherwise, Zhou *et al*.^18^ recruited participants from Wuhan for surveys before and after the lockdown was lifted revealing a slight improvement of individuals' mental health after the end of the lockdown. Until now, however, no study has explored the long‐term psychological consequences of the COVID‐19 outbreak and self‐isolation measures in Italian older adults.

Taking into account the above‐mentioned background, our main objective was to identify the impact of the pandemic and a long period (>1 month) of quarantine on the psychological and cognitive health status in a sample of Italian elderly, and determine which factors of the quarantine/self‐isolation were mostly associated with the occurrence of psychological symptoms. Particularly, we hypothesised that older people might experience psychological symptoms (e.g., depression and anxiety) and cognitive failures. Also, some variables related to the pandemic and the quarantine (e.g., the number of people they lived with and the number of outings in the last week) could be identified as risk factors for the development of psychological and cognitive symptoms, whereas personal resilience and adopting coping strategies might represent protective factors.

Moreover, we aimed to track longitudinal changes in their mental health status two months after the end of the lockdown and the factors associated with the development of PTS symptoms. We hypothesised a reduction of psychological symptoms after the end of the quarantine, but we expected to detect the presence of PTS symptoms.

## MATERIALS AND METHODS

### Participants

This study involved a subgroup of participants (individuals aged 60 or older) to a previous cross‐sectional survey performed to assess the psychological response of the Italian population during the quarantine/self‐isolation. In that study, an online questionnaire was created on a virtual platform of Google Moduli and shared in social networks (i.e., Facebook, Whatsapp, and social virtual groups) by friends, colleagues, and acquaintances via a snowball sampling strategy to recruit a large Italian sample of people living in different Italian regions. Study methods are extensively described in the above‐mentioned study.^19^

The data collection of the first wave (T0) was carried out from 4 April to 26 April 2020 (i.e., during the period in which quarantine was imposed by the Italian Government), whereas the second wave (T1) of data was obtained two months after the end of the quarantine and lockdown measures (i.e., from 20 July to 7 October). The study was approved by the Ethics Committee of University of Campania “Luigi Vanvitelli” and conformed to the principles embodied in the Declaration of Helsinki.

### Survey structure

The questionnaire included the following components.

1 An informed consent statement.

2 Queries about sociodemographic data and characteristics related to the pandemic and the quarantine/self‐isolation, namely age, gender, education, marital status, living status, household size, employment status, and previous psychiatric illnesses. Moreover, participants were asked to indicate how many days they have been in quarantine/self‐isolation, housing features (i.e., density, number of rooms, windows, and outdoor spaces), the number of people they lived with, the number of outings in the previous week. Additionally, they were asked to rate the frequency of feeling boredom, frustration, and fear of getting infected with COVID‐19, and to indicate if they had been admitted to hospital in the previous month and had been tested for COVID‐19, and if they had direct or indirect contacts with individuals with confirmed COVID‐19.

3 The Perceived Memory and Attentional Failures Questionnaire (PerMAFaQ) to assess subjective cognitive complaints. The tool consists of 9 items assessing perceived memory and attentional failures in everyday life activities performed at home (i.e., difficulty remembering the location of objects, difficulty remembering the content of a text, difficulty concentrating on the news of television or radio broadcasts, difficulty watching a movie until the end, difficulty concentrating while talking to someone else). Each item was to be rated on a 5‐point Likert scale ranging from 1 (never) to 5 (very often). The total score ranges from 5 to 45 with higher scores indicating a higher propensity to cognitive failures.

4 The Italian version of the Patient Health Questionnaire‐9 (PHQ‐9), a self‐report 9‐item inventory to evaluate symptoms of a major depressive episode according to the DSM‐5. Each item is rated on a 4‐point Likert scale ranging from 0 (not at all) to 3 (nearly every day). The total score ranges from 0 to 27; cut‐off points of 5, 10, 15, and 20 indicate mild, moderate, moderately severe, and severe levels of depressive symptoms.

5 The 7‐item Generalized Anxiety Disorder scale (GAD‐7) to assess the DSM‐IV symptoms for Generalized Anxiety Disorder was employed to evaluate anxiety. Each item is rated on a 4‐point Likert scale ranging from 0 (never) to 3 (nearly every day). The total score ranges from 0 to 21; GAD is indicated by a score equal to or greater than 10, whereas cut‐off points of 5, 10, and 15 indicate mild, moderate, and severe levels of anxiety.

6 The DSM‐5 Level 2‐Anger‐Adult measure (DSM‐5‐Anger), a 5‐item version of the PROMIS Anger Short Form, to assess the severity of anger symptoms during the past 7 days. Each item is rated on a 5‐point Likert scale ranging from 1 (never) to 5 (always). The total score ranges from 5 to 25; a higher total score indicates greater anger severity. The raw scores have to be converted to T‐scores which are interpreted in the following way: less than 55 = none to slight; 55.0–59.9 = mild; 60.0–69.9  = moderate; 70 and over = severe anger.

7 The Italian translation of the Brief Resilience Scale (BRS) to assess Resilience, which consists of 6 items. Each item is rated on a 5‐point Likert scale ranging from 1 (strongly disagree) to 5 (strongly agree). Items are formulated either positively (Items 1, 3, 5) or negatively (Items 2, 4, 6). The total score ranges from 6 to 30; a higher total score indicates the self‐referred ability to produce a positive adaptation response when facing adverse situations.

8 The Italian translation of the Coping Scale to assess the cognitive, emotional, and behavioural way of dealing with problems. This is a self‐report questionnaire consisting of 13 items. Each item is rated on a 4‐point Likert Scale ranging from 1 (not true about me) to 4 (mostly true about me). The total score ranges from 13 to 52; a higher total score indicates the use of adaptive coping strategies.

Finally, in the second wave (T1), the Italian version of the Impact of Event Scale‐Revised (IES‐R) was employed to evaluate the long‐term impact of the traumatic experience. This is a self‐report 22‐item scale comprising three subscales: intrusion (8 items), avoidance (8 items), and hyperarousal (6 items). Each item is rated on a 5‐point Likert scale ranging from 0 (not at all) to 4 (extremely) with higher scores indicating more severe post‐traumatic stress (PTS) symptomatology. The total score is interpreted in the following way: 0–8 = subclinical; 9–25 = mild; 26–43 = moderate; 44 and over = severe.

The references for the original tools are reported in Appendix S1 in the Supporting Information.

### Statistical analysis

Descriptive statistics were calculated for sociodemographic data, characteristics related to the pandemic and the quarantine/self‐isolation, and variables assessing cognitive failures, depressive symptoms, anxiety, anger, personal resilience, and the coping style of respondents.

Simple linear regression analyses were carried out to evaluate the relationships between depression, anxiety, and anger and the following: (i) sociodemographic characteristics such as age, sex, and educational level; (ii) characteristics related to the pandemic and the quarantine/self‐isolation such as duration of the quarantine/self‐isolation, number of people/children per house, number of rooms in the house, number of outings from home in a week, indirect contact with people affected by COVID‐19, fear of getting infected with COVID‐19; and (iii) coping style and personal resilience. Moreover, multiple regression analyses were performed entering mental health status as dependent variables along with the variables found to be significant predictors from the simple linear regression analyses.

The same analyses were performed to investigate the relationship between subjective cognitive failures and the above‐mentioned variables, with the addition of depression, anxiety, and anger as independent variables.

To investigate if and how resilience mediated the relationship between the fear of getting infected and mental health status, mediation analyses were carried out entering the fear of getting infected as the independent variable, mental health status variables as dependent variables, and resilience as the mediator.

The significance of direct, indirect, and total effects was evaluated by a bootstrapping procedure with 5000 samples with replacement from the full sample to construct bias‐corrected 95% confidence intervals (hereafter 95% CI; LL = lower level of the confidence interval, UL = upper level of confidence interval). This procedure was conducted with the SPSS Macro PROCESS.

To investigate longitudinal changes in participants' mental health status during (T0) and after (T1) the lockdown, we performed the Wilcoxon signed‐rank test. To control for type I errors, Bonferroni's correction for multiple comparisons was applied.

Finally, we carried out linear regression analysis to explore possible predictors of post‐traumatic stress disorder entering the IES‐R score as the dependent variable and variables about demographic data, characteristics related to the quarantine, and the mental health status provided during the first wave as independent variables.

The significance level was set at 0.05, and all statistical analyses were performed using SPSS Statistic 26.0.

## RESULTS

### First wave results

The sample of the first wave consisted of 334 elderly participants (196 females) (Table 1). The mean duration of quarantine/self‐isolation was 31.48 (SD: 4.7) days.

**Table 1:** Sociodemographic data of the sample of the first wave

Variables					
Age	60–69	70–79	80–89		
	279 (83.5%)	52 (15.6%)	3 (0.9%)		
Sex	Female	Male			
	196 (58.7%)	138 (41.3%)			
Level of education	Elementary	Middle school	High school	Degree and post‐degree	
	5 (1.5%)	26 (7.8%)	141 (42.2%)	162 (48.5%)	
Marital status	Married	Unmarried/maiden	Divorced/separated	Widower	
	217 (65%)	21 (6.3%)	69 (20.7%)	27 (8%)	
Number of people per household	Alone/1	2	3–5	6 or more	
	81 (24.3%)	118 (35.3%)	122 (36.5%)	13 (3.9%)	
Number of children per household	0	1–2	3–5	6 or more	
	298 (89.2%)	22 (6.6%)	9 (2.7%)	5 (1.5%)	
Number of rooms in the house	1–2	3	4	5 or more	
	21 (6.3%)	62 (18.6%)	96 (28.7%)	155 (46.4%)	
House with…	1 or more windows	Outdoor space (terrace, balcony, garden or shared courtyard)			
	21 (6.3%)	313 (93.7%)			
Employment status	Unemployed	Employed	Retired		
	38 (11.4%)	176 (52.6%)	120 (36%)		
Work modalities	Smart‐working	Office	No job		
	80 (24%)	37 (11%)	217 (65%)		
Duration of quarantine/self‐isolation	Mean (SD)	Median			
	31.48 (4.7)	30			
Number of outings in the last week	0	1–2	3–4	5 or more	
	70 (21%)	176 (52.7%)	50 (15%)	38 (11.3%)	
Being affected by COVID‐19	No	I do not answer	No (symptomatic but not tested by swab)	Yes (remitted)	
	320 (95.8%)	8 (2.4%)	5 (1.5%)	1 (0.3%)	
Direct contact with people affected by COVID‐19	(no answer)	No	Yes		
	9 (2.7%)	311 (93.1%)	14 (4.2%)		
Indirect contact with people affected by COVID‐19	(no answer)	No	Yes		
	5 (1.5%)	223 (66.8%)	106 (31.7%)		
Diagnosis of psychopathology	(no answer)	No	Yes		
	15 (4.5%)	311 (93.1%)	8 (2.4%)		
Boredom	Never	Sometimes	Often	Always	Mean (SD)
	108 (32.3%)	116 (34.7%)	63 (18.9%)	47 (14.1%)	2.15 (1.03)
Frustration	Never	Sometimes	Often	Always	Mean (SD)
	135 (40.4%)	87 (26%)	69 (20.7%)	43 (12.9%)	2.06 (1.06)
Fear of getting infected with COVID‐19.	Never	Sometimes	Often	Always	Mean (SD)
	66 (19.8%)	121 (36.2%)	90 (26.9%)	57 (17.1%)	2.41 (0.99)

Frequency (percentage); SD, standard deviation.

### Depression

Depressive symptomatology, evaluated by PHQ‐9, was absent or minimal in 153 (46%) respondents, mild in 125 (37%), moderate in 41 (12%), moderately severe in 13 (4%), and severe in 2 (1%) subjects. The mean score of the PHQ‐9 was 5.61 (SD: 4.23).

Simple regression analyses revealed that depression was significantly and negatively related to age, and scores on the BRS and Coping Scale, whereas a significant and positive relationship was found with the fear of getting infected (Table 2). To identify the most influential predictors of the PHQ‐9 score we carried out a multiple regression analysis where significant factors from simple regression analyses were entered as independent variables, particularly, entering age in block 1, scores on BRS and Coping Scale in block 2, and the fear of getting infected in block 3. This analysis revealed that a higher score on the PHQ‐9 was significantly related to younger age, a lower score on the BRS, and more fear of getting infected (Table 2).

**Table 2:** Results for regression analyses with PHQ‐9 score computed as dependent variable

		Simple regression analyses			95% confidence limits		Multiple regression analysis			95% confidence limits	
		*Beta*	*t*	*p*	*Lower*	*Upper*	*Beta*	*t*	*p*	*Lower*	*Upper*
Age		−0.166	−3.063	**0.002**	−0.255	−0.056	−0.135	−2.843	**0.005**	−0.214	−0.039
Sex		−0.081	−1.472	0.142	−1.612	0.232					
Level of education		0.045	0.830	0.407	−0.380	0.933					
Days of self‐isolation		0.053	0.968	0.334	−0.050	0.146					
Number of people per household		−0.057	1.031	0.303	−0.815	0.254					
Number of children per household		−0.035	−0.645	0.519	−1.140	0.577					
Number of rooms in the house		−0.089	−1.635	0.103	−0.887	0.082					
House density		0.013	0.225	0.822	−1.371	1.725					
Number of outings		0.015	0.277	0.782	−0.441	0.586					
Resilience		−0.451	−9.207	**<0.001**	−0.588	−0.381	−0.388	−7.862	**<0.001**	−0.522	−0.313
Coping		−0.205	−3.822	**<0.001**	−0.258	−0.083	‐	‐	**‐**	‐	‐
Infected people		0.097	1.783	0.076	−0.021	0.431					
Fear		0.323	6.216	**<0.001**	0.941	1.812	0.200	4.035	**<0.001**	0.437	1.267

Bold value indicates *P* values.

PHQ‐9, Patient Health Questionnaire‐9.

### Anxiety

An absence of anxious symptomatology as evaluated by the GAD‐7 was reported for 39% (131) of the respondents, whereas anxiety for 45% (151) was mild, for 11% (37) was moderate, and for 5% (15) was severe. The mean score of the GAD‐7 was 5.79 (SD: 4.05).

Simple regression analyses revealed that higher anxiety scores were significantly related to female gender, lower scores on the BRS and Coping Scale, and more fear of getting infected (Table 3). A multiple regression analysis entering sex in block 1, scores on the BRS and Coping Scale in block 2, and fear of getting infected in block 3 revealed that a higher score on the GAD‐7 was significantly related to a lower score on the BRS and more fear of getting infected (Table 3).

**Table 3:** Results for regression analyses with GAD‐7 score computed as dependent variable

		Simple regression analyses			95% confidence limits		Multiple regression analysis			95% confidence limits	
		*Beta*	*t*	*p*	*Lower*	*Upper*	*Beta*	*t*	*p*	*Lower*	*Upper*
Age		−0.107	−1.968	0.050	−0.193	0.000					
Sex		−0.139	−2.556	**0.011**	−2.021	−0.263	‐	‐	**‐**	‐	‐
Level of education		−0.073	−1.325	0.186	−1.052	0.205					
Days of self‐isolation		0.046	0.839	0.402	−0.054	0.134					
Number of people per household		0.035	0.632	0.528	−0.349	0.678					
Number of children per household		0.016	0.289	0.773	−0.703	0.945					
Number of rooms in the house		−0.060	−1.102	0.271	−0.726	0.205					
House density		0.071	1.276	0.203	−0.527	2.471					
Number of outings		−0.043	−0.782	0.435	−0.688	0.297					
Resilience		−0.460	−9.428	**<0.001**	−0.573	−0.375	−0.355	−7.475	**<0.001**	−0.462	−0.269
Coping		−0.236	−4.431	**<0.001**	−0.272	−0.105	‐	‐	**‐**	‐	‐
Infected people		0.060	1.089	0.277	−0.097	0.338					
Fear		0.448	9.135	**<0.001**	1.439	2.228	0.339	7.154	**<0.001**	1.006	1.769

Bold value indicates *P* values. GAD‐7, 7‐item Generalized Anxiety Disorder scale.

### Anger

Anger evaluated by the DSM‐5‐Anger was absent or minimal in 262 (78%) respondents, mild in 50 (15%), moderate in 19 (6%), and for 3 (1%) it was severe. The mean score of the DSM‐5‐Anger was 45.35 (SD: 9.27).

Simple regression analyses revealed that a higher anger score was significantly and negatively related to age, and scores on the BRS and Coping Scale, whereas a significant and positive relationship was found with the fear of getting infected and female gender (Table 4). Multiple regression analysis entering age and sex in block 1, scores on the BRS and Coping Scale in block 2, and fear of getting infected in block 3 revealed that a higher score on the anger scale was significantly related to female gender, a low score on the BRS, and more fear of getting infected (Table 4).

**Table 4:** Results for regression analyses with DSM‐5‐Anger score computed as dependent variable

		Simple regression analyses			95% confidence limits		Multiple regression analysis			95% confidence limits	
		*Beta*	*t*	*p*	*Lower*	*Upper*	*Beta*	*t*	*p*	*Lower*	*Upper*
Age		−0.111	−2.028	**0.043**	−0.448	−0.007	‐	‐	**‐**	‐	‐
Sex		−0.179	−3.313	**0.001**	−5.359	−1.366	−0.123	−2.550	**0.011**	−4.092	−0.528
Level of education		0.014	0.251	0.802	−1.257	1.625					
Days of self‐isolation		0.039	0.720	0.472	−0.136	0.293					
Number of people per household		0.057	1.036	0.301	−0.555	1.791					
Number of children per household		0.019	0.349	0.727	−1.549	2.218					
Number of rooms in the house		−0.054	−0.980	0.328	−1.595	0.534					
House density		0.051	0.924	0.356	−1.828	5.066					
Number of outings		−0.007	−0.131	0.895	1.202	1.051					
Resilience		−0.430	−8.684	**<0.001**	−1.244	−0.784	−0.321	−6.051	**<0.001**	−1.003	−0.511
Coping		−0.239	−4.477	**<0.001**	−0.626	−0.244	‐	‐	**‐**	‐	‐
Infected people		0.061	1.111	0.268	−0.217	0.779					
Fear		0.329	6.357	**<0.001**	2.128	4.035	0.211	4.213	**<0.001**	1.050	2.888

Bold value indicates *P* values. DSM‐5‐Anger, DSM‐5 Level 2‐Anger‐Adult measure.

### Mediation analyses

Taking into account the above‐mentioned results from multiple regression analyses, we designed a mediation model to test the mediator effect of resilience (BRS) on the relationship between the fear of getting infected and the mental health status variables. More fear of getting infected was related to poorer resilience (B = −1.145; *P* < 0.001). Subsequently, poorer resilience was related to more depressive symptoms (B = −0.419; *P* < 0.001), more anxiety (B = −0.371; *P* < 0.001), and more anger (B = −0.862; *P* < 0.001).

**Figure 1:**
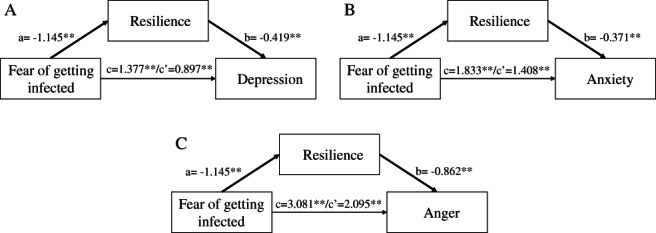
The mediation effects of resilience in the relationship between the fear of getting infected and (A) depression; (B) anxiety; (C) anger. In the arrow labels, a represents the effect of the fear of getting infected on resilience, b represents the effect of resilience on the mental health status variables, c is the total effect of the fear of getting infected on the mental health status variables, c' is the direct effect of the fear of getting infected on the mental health status variables controlling for the mediator. ** *P* < 0.001.

The bias‐corrected 95% CI based on 5000 bootstrap samples revealed that the indirect effects of the fear of getting infected on depressive symptoms (estimate effect: 0.480; 95% CI: 0.266–0.735), anxiety (estimate effect: 0.425; 95% CI: 0.248–0.625), and anger (estimate effect: 0.986; 95% CI: 0.558–1.472) through resilience abilities were all significant, indicating mediation of resilience for all relationships between the fear of getting infected and mental health status (Fig. 1).

### Subjective cognitive failures

Subjective cognitive failures occurred in 32% of the respondents, and the mean score on the PerMAFaQ was 18.32 (SD: 6.43). The most frequent cognitive failures were about ‘remembering where you left things’ (47%) and ‘forgetting the reason why you went from one part of the house to another’ (50.9%), whereas ‘to have trouble focusing while talking to someone’ (21%) was the less frequent cognitive complaint (Appendix S2). Simple regression analyses revealed that the PerMAFaQ score was significantly and negatively related to scores on the BRS and Coping Scale, and significantly and positively related to female gender, scores on the PHQ‐9, GAD‐7, and DSM‐5‐Anger, and fear of getting infected (Table 5). A multiple regression analysis entering sex in block 1, depression, anxiety, and anger scores in block 2, scores on the BRS and Coping Scale in block 3, and fear of getting infected in block 4 revealed that a higher score on the PerMAFaQ was significantly related to female gender, a lower score on the BRS, and higher scores on the PHQ‐9 and DSM‐5‐Anger (Table 5).

**Table 5:** Results for regression analyses with PerMAFaQ score computed as dependent variable

		Simple regression analyses			95% confidence limits		Multiple regression analysis			95% confidence limits	
		*Beta*	*t*	*p*	*Lower*	*Upper*	*Beta*	*t*	*p*	*Lower*	*Upper*
Age		−0.058	−1.062	0.289	−0.236	0.071					
Sex		−0.169	−3.132	**0.002**	−3.595	−0.821	−0.089	−2.025	**0.044**	−2.280	−0.033
Level of education		0.020	0.368	0.713	−0.812	1.186					
Days of self‐isolation		−0.065	−1.181	0.238	−0.237	0.059					
Number of people per household		−0.047	−0.863	0.389	−1.171	0.457					
Number of children per household		0.032	0.586	0.558	−0.917	1.695					
Number of rooms in the house		−0.040	−0.737	0.462	−1.016	0.462					
House density		−0.086	−1.549	0.122	−4.198	0.499					
Number of outings		−0.055	−1.006	0.315	−1.179	0.381					
Resilience		−0.425	−8.562	**<0.001**	−0.855	−0.535	−0.172	−3.503	**0.001**	−0.439	−0.123
Coping		−0.135	−2.477	**0.014**	−0.305	−0.035	‐	‐	‐	‐	‐
Infected people		0.085	1.558	0.120	−0.072	0.618					
Fear		0.235	4.412	**<0.001**	0.846	2.207					
PHQ‐9		0.574	12.769	**<0.001**	0.739	1.008	0.370	6.186	**<0.001**	0.384	0.742
GAD‐7		0.419	8.406	**<0.001**	0.509	0.820	‐	‐	‐	‐	‐
DSM‐5‐Anger		0.515	10.960	**<0.001**	0.293	0.422	0.177	2.966	**0.003**	0.041	0.205

Bold value indicates *P* values.

PerMAFaQ, Perceived Memory and Attentional Failures Questionnaire; PHQ‐9, Patient Health Questionnaire‐9; GAD‐7, 7‐item Generalized Anxiety Disorder scale; DSM‐5‐Anger, DSM‐5 Level 2‐Anger‐Adult measure.

### Second wave results

Fifty participants (29 females) completed the second wave survey. The mean age was 64.94 (SD: 4.23).

### Longitudinal changes in mental health status

No significant difference was found on Boredom, Frustration, and Fear of getting infected items (Table 6).

**Table 6:** Comparison of the mental health status scores during and after the quarantine

	During the quarantine (T0)	After the quarantine (T1)		
	*Mean ± SD*	*Mean ± SD*	*Z*	*p*
Boredom	2.08 ± 0.97	1.90 ± 0.89	−1.235	0.217
Frustration	2.12 ± 1.08	1.98 ± 0.94	−0.862	0.388
Fear of getting infected	2.48 ± 0.86	2.24 ± 0.89	−1.935	0.053
PHQ‐9	6.04 ± 4.26	6.56 ± 4.77	−0.883	0.377
GAD‐7	5.12 ± 3.91	6.10 ± 4.34	−1.995	0.046
DSM‐5‐Anger	50.42 ± 9.37	51.91 ± 9.74	−1.406	0.160
PerMAFaQ	19.08 ± 6.58	19.38 ± 6.61	−0.509	0.611
BRS	20.22 ± 4.24	20.96 ± 3.89	−1.418	0.156
Coping scale	33.94 ± 5.57	32.98 ± 5.38	−1.984	0.047

SD, standard deviation; BRS, Brief Resilience Scale; GAD‐7, 7‐item Generalized Anxiety Disorder scale; DSM‐5‐Anger, DSM‐5 Level 2‐Anger‐Adult measure; PHQ‐9, Patient Health Questionnaire‐9; PerMAFaQ, Perceived Memory and Attentional Failures Questionnaire.

Significant difference after Bonferroni correction (0.05/9 = 0.005).

Moreover, no significant difference was found for mental health (i.e., depression, anxiety, and anger) and cognitive status, nor on resilience and coping scores after Bonferroni's correction (Table 6).

### Post‐traumatic stress disorder

Post‐traumatic stress disorder, evaluated by the IES‐R, was subclinical or absent for 8% of respondents, whereas for 72% it was mild, for 16% it was moderate, and for 4% it was severe. The mean score of IES‐R was 19.90 (SD: 10.55).

Simple regression analyses revealed that the development of post‐traumatic stress symptoms at T1 was significantly and positively related to more fear of getting infected, more cognitive failures, and more severe depression, anxiety, and anger symptoms measured at T0 (Table 7). To identify the most influential predictors of the IES‐R score we carried out a multiple regression analysis where significant factors from the simple regression analyses were entered as independent variables, particularly, entering depression, anxiety, anger, and cognitive failures in block 1 and the fear of getting infected in block 2. This analysis revealed that a higher score on the IES‐R at T1 was significantly related to more severe depressive symptoms evaluated through the PHQ‐9 at T0 (Table 7).

**Table 7:** Results for regression analyses with IES‐R score computed as dependent variable

		Simple regression analyses			95% confidence limits		Multiple regression analysis			95% confidence limits	
		*Beta*	*t*	*p*	*Lower*	*Upper*	*Beta*	*t*	*p*	*Lower*	*Upper*
Age		−0.005	−0.033	0.974	−0.736	0.712					
Sex		0.015	0.105	0.917	−5.820	6.461					
Level of education		0.097	0.672	0.505	−0.779	1.562					
Days of self‐isolation		−0.074	−0.516	0.608	−0.270	0.159					
Number of people per household		0.225	1.584	0.120	−0.604	5.079					
Number of children per household		0.057	0.389	0.699	−5.874	8.694					
Number of rooms in the house		−0.032	−0.218	0.828	−2.620	2.106					
Number of outings		0.003	0.019	0.985	−1.670	1.702					
Resilience at T0		−0.205	−1.453	0.153	−1.218	0.196					
Coping at T0		−0.170	−1.198	0.237	−0.865	0.219					
Fear at T0		0.431	3.312	**0.002**	2.073	8.476	‐	‐	**‐**	‐	‐
PHQ‐9 at T0		0.532	4.353	**<0.001**	0.709	1.926	0.532	4.353	**<0.001**	0.709	1.926
GAD‐7 at T0		0.507	4.081	**<0.001**	0.696	2.047	‐	‐	**‐**	‐	‐
DSM‐5‐Anger at T0		0.436	3.360	**0.002**	0.197	0.785	‐	‐	**‐**	‐	‐
PerMAFaQ at T0		0.280	2.021	**0.049**	0.002	0.896	‐	‐	**‐**	‐	

Bold value indicates *P* values.

IES‐R, Impact of Event Scale‐Revised; PHQ‐9, Patient Health Questionnaire‐9; GAD‐7, 7‐item Generalized Anxiety Disorder scale; DSM‐5‐Anger, DSM‐5 Level 2‐Anger‐Adult measure; PerMAFaQ, Perceived Memory and Attentional Failures Questionnaire; T0, time of first wave.

## DISCUSSION

The present study investigated the mental health status of an Italian elderly sample during COVID‐19 quarantine/self‐isolation, revealing that 54% of the elderly experienced mild depressive symptoms and 17% reported moderate to severe depression, whereas the 45% of the participants reported mild anxiety, 11% had moderate anxiety, and 5% reported severe anxiety. As for anger, 22% of respondents felt angry during this period. These prevalence rates seem to be in line with those provided by Meng *et al*.,^16^ who reported that 37.1% of a Chinese elderly sample experienced depression and anxiety during COVID‐19. These results indicate that elderly people are vulnerable to experiencing psychological symptoms, and thus need online psychological interventions to reduce the long‐term consequences on mental health caused by the crisis.

When investigating the factors associated with psychological symptoms, we found an association of more severe depressive symptoms with younger age, poorer resilience and coping strategies, and more fear of getting infected. Moreover, more anxiety was associated with female gender, poorer resilience and coping strategies, and more fear of getting infected; whereas higher anger levels were related to younger age, female gender, poorer resilience and coping strategies, and more fear of getting infected.

The above‐mentioned findings support previous results of a strong relationship between psychological symptoms and the female gender both in the elderly^16^ and in the general population.^9^, ^10^, ^19^, ^20^, ^21^ Furthermore, we found that younger age was associated with more depressive symptoms and more anger. Taking into account that in our sample we enrolled only respondents who were at least 60 years old, in this range a younger age was related to altered mental status since the stay‐at‐home restrictions and the quarantine may impact daily routine and habits of ‘young elderly’ rather than of ‘older’ ones.

The most influential predictors of mental health status from the multiple regression analyses were resilience abilities and the fear of getting infected. More specifically, poorer resilience abilities and more fear of getting infected were related to more severe depression, anxiety, and anger. The fear of COVID‐19, probably due to its novelty and the uncertainties about the course and end of the pandemic has led to the use of the term ‘coronaphobia’.^22^ Several psychological vulnerability factors such as perceived vulnerability to disease, a tendency to worry, intolerance of uncertainty, and other individual variables may be predictive factors for coronaphobia.^23^ In this regard, our findings of a strong relationship between fear of getting infected and the mental health status (i.e., depressive symptoms, anxiety, and anger) may suggest that perceived vulnerability to COVID‐19 plays a crucial role in the development of psychological symptoms in elderly.

We also found that resilience, rather than adaptive coping strategies, seems to be a protective factor amongst the development of psychological symptoms in advanced age confirming the findings of a previous study^24^ where personal resilience emerged as a crucial factor of psychological functioning during the COVID‐19 pandemic. It was observed that resilience abilities counter the detrimental effects of various demographic and health‐related variables attenuating their impact on mental health.^24^

To evaluate the protective effect of resilience abilities on elderly mental health status against the fear of getting infected, we performed several mediation analyses. We found that resilience was a significant mediator in the relationship between the fear of getting infected and the mental health status of elderly. These results further support the conceptualization of resilience as a personal trait that protects individuals against the impact of traumatic and stressful life events,^25^ suggesting the implementation of psychosocial and cognitive‐training interventions to enhance the resilience^26^ of individuals who are at risk for stress‐induced psychological symptoms.

Finally, 32% of the respondents reported subjective cognitive failures. These prevalence rates seem to be slightly higher than those reported in a previous study conducted during the COVID‐19 quarantine for the Italian general population (27.5%^19^) and support previous findings of an age‐related decline in subjective cognitive functioning which could start at the age of 50 and steadily increase afterward.^27^ We identified female gender, poorer resilience, and more severe depressive and anger symptoms as factors associated with subjective cognitive failures. These findings confirm an impact of mental health status on complaints of attentive and memory difficulties in elderly people as reported in our previous study on the Italian general population^19^ and are in line with studies on the relationship between depression and cognitive deficits^28^ in elderly people. Taken together, our results strengthen the idea of the need to support vulnerable groups of elderly to reduce possible long‐term cognitive consequences due to self‐isolation/quarantine.

Moreover, we tracked longitudinal changes in participants' mental health two months after the end of the lockdown. We did not find any significant difference in mental health scores reported during and after the quarantine. This result further supports the idea of long‐term psychological consequences due to the outbreak since no reduction was observed in depressive, anxiety, and anger scores despite the hot phases of the pandemic were elapsed.

Otherwise, the slight increase in anxiety score, although not significant after the correction application, may suggest that elderly subjects, as a vulnerable group, perceived as dangerous the end of self‐restriction measures since this could represent a risk for the virus spread and thus for their health.

As for PTS symptoms, 20% of participants reported moderate to severe symptomatology at follow‐up evaluation, and we found that depressive symptoms turned out to be the most influent predictive factor in the development of PTS symptoms. Taking into account that significant PTS symptoms can evolve into PTS disorder and that its symptoms tend to be related to significant physical and psychological impairments,^29^ it is essential to develop timely intervention programs to reduce the impact of traumatic experience on individuals' mental health and to identify early people at high risk of developing PTS disorder.

Accordingly, the rise in psychological symptoms observed throughout the COVID‐19 outbreak was unlikely to be due to seasonality or year‐to‐year variation,^30^ and should be taken into account since the concomitant presence of severe psychological symptoms and traumatic life events should be considered a warning sign for suicidal behaviour in elderly and more vulnerable groups.^31^, ^32^

The present study is characterised by some limitations: (i) a snowball strategy not balanced on a‐priori basis was employed to recruit the respondents and this fact could limit generalization of the results since the representativeness of the sample could not be guaranteed; (ii) the limited number of participants recruited after the end of self‐restriction measures, primarily due to the difficulty in recruiting older people through online surveys; (iii) the use of a unique item and not a validated scale to evaluate the fear of getting infected. Thus, our results should be confirmed by further studies.

In conclusion, the fear of getting infected, probably due to a perceived vulnerability to disease, seems to play a crucial role in the development of psychological symptoms in the elderly. Nevertheless, resilience seems to reduce psychological symptoms, mediating the impact of fear. Finally, our findings from the longitudinal analysis suggest the presence of long‐term psychological consequences and the possible risk of developing PTS disorder in elderly.

Therefore, targeted interventions to reduce psychological symptoms, especially in elderly who are most at risk for COVID‐19, are needed in order to mitigate possible long‐term consequences such as more severe cognitive impairment and PTS disorder.

## Author contributions

G.M. contributed substantially to the conception or design of the work, carried out the analysis and interpretation of the work's data and wrote the draft; I.B., A.B., N.D.C., M.C., and R.N. contributed substantially to the execution of the study and carried out the analysis and interpretation of the work's data; G.S. critically reviewed it for its important intellectual content.

All authors have approved the final article.

## Supporting information


**Appendix S1.** References of tools
**Appendix S2.** Descriptive of items included in the Perceived Memory and Attentional Failures Questionnaire
