# Supplementary material for: Mental health status of Italian elderly subjects during and after quarantine for the COVID‐19 pandemic: a cross‐sectional and longitudinal study
Source: Psychogeriatrics. 2021 May 6;21(4):540–51. doi: 10.1111/psyg.12703 (PMC8242477; doi:10.1111/psyg.12703)
Supplement: Supplementary file 1 — Appendix S1. References of tools Appendix S2. Descriptive of items included in the Perceived Memory and Attentional Failures Questionnaire [file PSYG-21-540-s001.zip › PSYG_12703_Supplemental Material 1_reference tools.docx]

**Supplemental Material 1. References of tools**

*Mental health status*

Anxiety symptoms were assessed using the 7-item Generalized Anxiety Disorder scale (GAD-7; [1]) assessing the DSM-IV symptoms for Generalized Anxiety Disorder [2].

1. Spitzer RL, Kroenke K, Williams JB, et al. A brief measure for assessing generalized anxiety disorder: the GAD-7. Arch Intern Med. 2006; 22;166(10):1092-7. doi: 10.1001/archinte.166.10.1092
2. American Psychiatric Association. Diagnostic and Statistical Manual of Mental Disorders (4th ed.).1994. Washington, DC: American Psychiatric Association.

Anger was assessed using the DSM-5 Level 2-Anger-Adult measure [3], a 5-item version of the PROMIS Anger Short Form assessing severity of individual’s anger during the past 7 days (DSM-5-Anger). For the Italian version of the scale, the code is Code IT/CPSZ/03 DSM5 Rabbia Livello 2 Adulti and is available in https://www.psicologozatelli.it/dsm5-test-in-italiano/

1. American Psychiatric Association. Diagnostic and statistical manual of mental disorders (5th ed.). 2013. Washington, DC: American Psychiatric Association.

Depressive symptoms were evaluated using the Italian version of the Patient Health Questionnaire-9 (PHQ-9; [4,5]),

1. Kroenke K, Spitzer RL, Williams JB. The PHQ-9: validity of a brief depression severity measure. J Gen Intern Med. 2001;16(9):606-13. Doi: 10.1046/j.1525-1497.2001.016009606.x
2. Mazzotti E, Fassone G, Picardi A, et al. The Patient Health Questionnaire (PHQ) for the screening of psychiatric disorders: a validation study versus the Structured Clinical Interview for DSM-IV axis I (SCID-I). Ital J Psychopathol 2003;9: 235-42.

Resilience

Individual response to stressful situations was assessed by means of the Brief Resilience Scale (BRS, [6])

1. Smith, Bruce W., Dalen et al. The brief resilience scale: assessing the ability to bounce back. International journal of behavioral medicine. 2008;15(3):194-200. doi: 10.1080/10705500802222972

Coping style

The cognitive, emotional, and behavioral way for dealing with problems were assessed using the Coping Scale [7].

1. Hamby S, Grych J, Banyard VL. Life paths measurement packet. 2013. Sewanee, TN: Life Paths Research Program.

Post-traumatic Stress Disorder

The post-traumatic stress disorder was evaluated using the Impact of Event Scale-Revised [8,9].

8. Sterling M. The Impact of Event Scale (IES). Aust J Physiother. 2008;54(1):78. doi: 10.1016/s0004-9514(08)70074-6. PMID: 18567188.

9. Craparo G, Faraci P, Rotondo G, Gori A. The Impact of Event Scale - Revised: psychometric properties of the Italian version in a sample of flood victims. Neuropsychiatr Dis Treat. 2013;9:1427-1432. doi:10.2147/NDT.S51793
