# Supplementary material for: Mental health status of Italian elderly subjects during and after quarantine for the COVID‐19 pandemic: a cross‐sectional and longitudinal study
Source: Psychogeriatrics. 2021 May 6;21(4):540–51. doi: 10.1111/psyg.12703 (PMC8242477; doi:10.1111/psyg.12703)
Supplement: Supplementary file 1 — Appendix S1. References of tools Appendix S2. Descriptive of items included in the Perceived Memory and Attentional Failures Questionnaire [file PSYG-21-540-s001.zip › PSYG_12703_Supplemental Material 2.docx]

**Supplementary Material 2. Descriptive of items included in the Perceived Memory and Attentional Failures Questionnaire**

| Items | Never | Rarely | Sometimes | Often | Always | Sometimes to Always | |
| --- | --- | --- | --- | --- | --- | --- | --- |
| In the period of the quarantine/self-isolation… |  |  |  |  |  |  | |
| 1. Do you have trouble remembering where you left your things (e.g. glasses, keys, mobile phone)? | 66 (19.8%) | 111 (33.2%) | 114 (34.1%) | 33 (9.9%) | 10 (3%) | 47% | |
| 2. Do you have trouble remembering the contents of newspapers, newscasts s and newsletter? | 127 (38%) | 114 (34.2%) | 86 (25.7%) | 0 (0%) | 7 (2.1%) | 27.8% | |
| 3. Do you have trouble focusing on the news you hear on television or radio broadcasts? | 149 (44.6%) | 98 (29.3%) | 71 (21.3%) | 14 (4.2%) | 2 (0.6%) | 26.1% | |
| 4. Do you have trouble watching a movie from start to the end? | 154 (46.1%) | 85 (25.4%) | 67 (20.1%) | 19 (5.7%) | 9 (2.7%) | 28.5% | |
| 5. Do you have trouble focusing while talking to someone? | 175 (52.4%) | 89 (26.6%) | 64 (19.2%) | 4 (1.2%) | 2 (0.6%) | 21% | |
| 6. Do you have trouble focusing while reading a newspaper or a book? | 127 (38%) | 117 (35%) | 64 (19.2%) | 23 (6.9%) | 3 (0.9%) | 27% | |
| 7. Do you have trouble doing something at home because you end up doing something else without even realizing it? | 153 (45.8%) | 92 (27.5%) | 60 (18.0%) | 17 (5.1%) | 12 (3.6%) | 26.7% |  |
| 8. Has it happened to you to forget the reason why you went from one part of the house to another? | 72 (21.6%) | 92 (27.5%) | 128 (38.3%) | 31 (9.3%) | 11 (3.3%) | 50.9% | |
| 9. Has it accidentally happened to you to leave the light or the television on in a room? | 116 (34.7%) | 108 (32.3%) | 89 (26.7%) | 11 (3.3%) | 10 (3%) | 33% | |
|  |  |  |  |  |  | Mean 32% | |
